# Immunologically Adaptive Endovascular Devices: Integrating Thrombo-Inflammation, Biomaterials Design, and Artificial Intelligence for Precision Cardiovascular Intervention

**DOI:** 10.3390/ijms27083493

**Published:** 2026-04-14

**Authors:** Rasit Dinc, Nurittin Ardic

**Affiliations:** 1 INVAMED Medical Innovation Institute, New York, NY 10007, USA; 2 Med-International UK Health Agency Ltd., Nuneaton CV11 6LT, Warwickshire, UK; nurittinardic@yahoo.com

**Keywords:** endovascular devices, thrombo-inflammation, extracellular vesicles, artificial intelligence, immune–device interaction, precision cardiovascular intervention

## Abstract

Endovascular therapies have transformed cardiovascular medicine, yet restenosis, thrombosis, and device failure remain common and poorly predictable complications. Increasing evidence suggests that immunothrombotic processes critically shape vascular recovery after device implantation. This includes neutrophil extracellular trap (NET) formation, innate immune polarization, and endothelial damage responses. Concurrently, advances in artificial intelligence (AI) are increasingly enabling continuous multimodal monitoring and adaptive clinical decision-making throughout the medical device life cycle. Here, we propose the concept of immunologically adaptive endovascular devices: a closed-loop paradigm in which patient immune status informs device selection, device–tissue interactions are interpreted via mechanistic biomarkers, and real-world monitoring dynamically updates risk and management. The study introduces (i) an immune–device interaction phenotype taxonomy linking device design features to measurable thrombo-inflammatory trajectories, (ii) a mechanistic framework defining interface signaling processes that enhance or resolve NET-driven responses, (iii) a minimum evidence model encompassing preclinical testing, clinical validation, and post-market surveillance, and (iv) a reference AI architecture for risk prediction, drift detection, and safety monitoring. This study also outlined testable predictions and a translational roadmap toward precision endovascular intervention and next-generation adaptive cardiovascular devices.

## 1. Introduction: Why Endovascular Outcomes Remain Unpredictable

Endovascular therapies have transformed cardiovascular and peripheral vascular care by enabling minimally invasive treatment of stenotic, aneurysmal, and thrombotic diseases. Advances in stent architecture, drug-eluting technologies, embolization systems, and bioabsorbable scaffolds have significantly expanded catheter-based capabilities over the past three decades [1,2,3,4]. However, restenosis, thrombosis, and device failure remain clinical challenges limiting long-term outcomes despite these technological advances. Restenosis rates of 5–20% in drug-eluting stents and as high as 30–40% in complex peripheral interventions continue to trigger re-intervention [3,5,6,7]. Clinical variability among patient populations suggests that vascular healing after implantation is governed by mechanisms beyond mechanical design and pharmacological coating strategies.

Historically, device development and selection have been driven by pharmacological strategies aimed at suppressing neointimal proliferation, along with engineering priorities such as radial strength, applicability, scaffold geometry, and flow effects [1,4,8,9]. While these approaches have significantly reduced early complications, they do not fully account for the complex biological responses triggered by vascular injury and foreign body exposure. Increasing evidence suggests that the vascular response to implanted devices is primarily shaped by thrombo-inflammatory processes involving innate immune activation, endothelial dysfunction, and platelet–leukocyte interactions [10,11,12]. These biological processes create significant heterogeneity in post-implantation healing trajectories and contribute to different clinical outcomes even when the same devices are used.

Among the thrombo-inflammatory mediators, neutrophil extracellular traps (NETs) have emerged as key factors linking innate immune activation to thrombosis and vascular injury. NETs, extracellular chromatin structures released by activated neutrophils, were initially identified as antimicrobial defenses [13]. They are now recognized as central regulators of cardiovascular pathology, including atherosclerosis, myocardial infarction, ischemic stroke, and venous thromboembolism [14,15,16,17,18]. NETs provide a scaffold for platelet adhesion and fibrin deposition, enhance inflammatory signaling, and have been implicated in device-associated thrombosis and restenosis [19]. A comprehensive review of NETs in homeostasis and disease [20] and detailed analyses of NETs in cardiovascular and aortic pathology [15] further highlight their importance. Our recent study demonstrated that NETs affect biocompatibility, degradation kinetics, and device performance in bioabsorbable vascular scaffolds [21].

A second converging trend is the maturation of extracellular vesicle (EV) biology in cardiovascular disease. EVs, including exosomes and microvesicles, facilitate intercellular communication by transferring proteins, lipids, and regulatory RNAs between vascular cells, platelets, and immune populations [22,23,24]. Platelet-derived EVs spread procoagulant surfaces beyond the initial activation site [25], while EVs from endothelial and immune cells modulate neutrophil function and thrombo-inflammatory cascades [26,27,28]. We have previously investigated how EVs can serve as biological templates for next-generation drug-eluting cardiovascular devices [29]. The emerging interaction between EVs and NETs suggests that these pathways may form amplification loops that can exacerbate or resolve thrombo-inflammatory processes depending on the molecular context [26,30].

Concurrently, advances in artificial intelligence (AI) and real-world data infrastructure are reshaping how medical devices can be monitored after implantation. Instead of relying on fixed follow-up programs, machine learning models can integrate imaging, laboratory biomarkers, and electronic health records to detect subtle signals of device failure and guide adaptive clinical decision-making processes [31,32,33,34,35]. Analyses of FDA-approved AI-enabled devices [36] and studies on pre-defined change control schemes for adaptive algorithms [37] demonstrate how regulatory frameworks are evolving to accommodate life cycle monitoring. Separately, we proposed how AI-driven decision-making can guide intravascular device selection in aortic disease [38].

Taken together, these developments motivate a new conceptual model: endovascular intervention is treated as a dynamic biotechnological system where immune status interacts with device characteristics to shape outcomes, and AI-enabled monitoring creates a feedback loop for adaptive management. In this study, we introduce the concept of immunologically adaptive endovascular devices, a paradigm that integrates thrombo-inflammatory biology with device engineering and computational monitoring. We propose an immune–device interaction phenotype taxonomy, define the mechanistic pathways governing the immune–device interface, outline a life cycle evidence framework, and present testable predictions with a translational roadmap toward precision endovascular medicine.

The aim of this review is to define a translational framework for immunologically adaptive endovascular devices by integrating thrombo-inflammatory biology, biomaterial design, and AI-enabled monitoring. Specifically, we aim to clarify how immune system–device interaction phenotypes emerge, how they can be measured, how they can contribute to device-specific risk stratification, and how they can be incorporated into life cycle evidence generation and closed-loop clinical monitoring.

## 2. Immune–Device Interface as a Determinant of Outcome

This section defines the immune system–device interface as the biological starting point from which thrombo-inflammatory processes arise.

Implantation of endovascular devices creates an interface between engineered materials and host vascular tissues, triggering an organized wound healing response. Classical biomaterial frameworks define the sequential stages of protein adsorption, acute inflammation, chronic inflammation, granulation tissue formation, and fibrous encapsulation [31,39]. However, recent advances in vascular immunology reveal a more nuanced picture, particularly where thrombo-inflammation determines whether the vessel will adaptively heal or progress toward restenosis and thrombosis.

Vascular injury exposes the subendothelial matrix, leading to rapid platelet adhesion and activation. Platelets then coordinate leukocyte recruitment via chemokines and adhesion pathways, linking hemostasis with innate immunity in a process called immunothrombosis [10,12]. Under physiological conditions, this response supports injury repair and the host defense; however, when dysregulated, it promotes thrombus formation, vascular occlusion, and maladaptive remodeling [11,40]. The role of hemodynamic shear stress in regulating endothelial function and leukocyte adhesion further shapes local inflammatory microenvironments near device supports and disordered surfaces [8,9].

Neutrophils are early responders to vascular injury and are increasingly recognized as drivers of thrombo-inflammatory amplification. NETs, chromatin networks released during neutrophil activation, were first characterized as antimicrobial structures [13] and subsequently shown to promote deep vein thrombosis [41,42] and arterial thrombus formation [17]. In the cardiovascular environment, NETs contribute to atherosclerosis progression, plaque destabilization, and ischemic damage [14,15]. Coronary NET load and DNase activity have been shown to predict infarct size in acute coronary syndrome with ST elevation [19]. In the context of implanted devices, biomaterial surfaces modulate neutrophil activation through surface chemistry, coating composition, and degradation products [21,39]. Our recent analysis has shown that NETs affect biocompatibility and degradation kinetics in bioabsorbable vascular scaffolds [21], while broader reviews highlight NETs as therapeutic targets in cardiovascular and aortic diseases [15,20].

The immunological outcomes of device implantation are not only determined by host biology but also shaped by device design. Pathology and preclinical studies in drug-eluting stents highlight that polymer coatings, drug release kinetics, and surface topography affect endothelial regeneration and neointimal growth [1,3,43]. Delayed healing and incomplete endothelialization remain key factors in delayed stent thrombosis and neoatherosclerosis [1,43]. Drug-coated balloons present a different set of variables affecting outcomes such as drug delivery efficiency, adjuvant effects, and vessel wall contact time [5,6,7]. Bioabsorbable scaffolds add temporal complexity with degradation products interacting with immune cells and vascular tissues for months to years [4,21].

Monocytes and macrophages contribute significantly to long-term remodeling after implantation. Macrophage polarization and phenotypic plasticity influence whether the vessel wall progresses toward resolution (M2-like repair) or persistent inflammation and neointimal hyperplasia (M1-like persistence) [11,44]. Feedback loops between macrophages, neutrophils, and endothelial cells can either amplify or resolve inflammatory responses in the vascular wall [11,12].

Collectively, the immune–device interface functions as a checkpoint for endovascular outcomes. Therefore, device performance should be conceptualized not as a static property of materials and geometry, but as an emerging biological response shaped by thrombo-inflammatory dynamics. In the following section, we examine how intercellular signaling mechanisms, particularly those involving extracellular vesicles and NET-mediated pathways, can further modulate these dynamics at the device surface.

## 3. Interface Signaling Mechanisms: Extracellular Vesicles and NET-Linked Thrombo-Inflammation

After defining the interface between the immune system and the device, we next examine the intercellular signaling mechanisms, specifically EV-NET interactions, that can amplify or decode these responses.

Beyond direct cell–material interactions, vascular injury initiates complex intercellular communication that can enhance or resolve thrombo-inflammation. Extracellular vesicles (EVs), including exosomes (30–150 nm) and microvesicles (100–1000 nm), are released by endothelial cells, platelets, leukocytes, and smooth muscle cells in response to activation, stress, and injury [23]. EVs carry biologically active cargo (proteins, lipids, microRNAs) that enable long-range signaling in damaged vascular environments [24,45]. A critical systematic review of EV clinical trials has highlighted both the therapeutic promise and translational challenges of this rapidly expanding field [46].

In thrombotic environments, platelet-derived EVs are particularly important. Studies on platelet EV biology highlight that vesicles can extend their coagulation-promoting surfaces and signal loads beyond the immediate site of platelet activation [25]. Platelet EVs interact with leukocytes and vascular cells to enhance adhesion and promote thrombus expansion [45,47]. Endothelial-derived EVs similarly contribute to thrombo-inflammatory cascades by transporting tissue factor, adhesion molecules, and inflammatory mediators [24]. The interaction between neutrophils, platelets, and extracellular vesicles in thrombopathic settings has been studied in detail [26].

**Figure 1:**
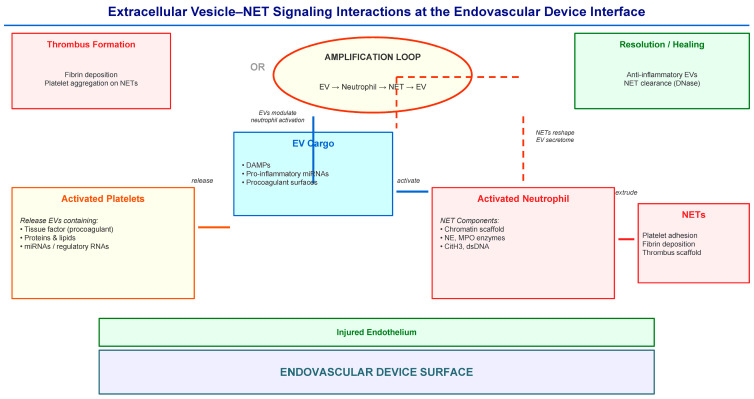
Extracellular vesicle–NET signaling interactions at the endovascular device interface. Vascular injury and biomaterial contact stimulate platelet activation, leukocyte recruitment, and extracellular vesicle release. EV contents, including proteins, lipids, and regulatory RNAs, can modulate neutrophil activation and inflammatory signaling pathways. Activated neutrophils release neutrophil extracellular traps that serve as scaffolds for platelet adhesion and clotting factors. NET-associated enzymes and inflammatory mediators can further reshape the vesicle microenvironment, creating a potential amplification loop that enhances thrombo-inflammatory signaling around implanted devices. Solid arrows indicate direct signalling pathways. Dashed arrows indicate the feedback loop in which NET components modify EV cargo, creating a self-amplifying thromboinflammatory cycle.

How can EVs intersect with NET-driven immunothrombosis? The most plausible synthesis is that EVs participate in thrombo-inflammatory networks involving neutrophils, platelets, and endothelial cells; NET formation is a central node in these networks. EVs carry cargo that can modulate neutrophil activation, including inflammatory cytokines, reactive oxygen species, and toll-like receptor ligands [26]. NET-associated enzymes (e.g., neutrophil elastase and myeloperoxidase) can reshape the local inflammatory secretome and vesicle environment in NET-rich environments, directing signaling toward coagulation-promoting and danger-associated patterns [17,20]. This bidirectional relationship creates a potential amplification loop: device-triggered EV release prepares neutrophils for NETosis, while NET components alter the local EV secretome toward a more prothrombotic phenotype. The proposed signaling interactions between extracellular vesicles and NET-induced thrombo-inflammation are schematically summarized in Figure 1.

Preclinical studies suggest that platelet and endothelial-derived EVs can intensify thrombo-inflammatory signaling and interact with neutrophil activation pathways; clinical trials increasingly support the use of circulating EV and NET-related markers as indicators of thrombotic activity and vascular damage.

From an engineering perspective, this EV-NET-associated signaling layer is important because biomaterial surfaces can shape cellular activation and therefore influence the upstream release and composition of EVs [21,39,48]. Polymer coatings and their degradation products can modulate EV release from endothelial and immune cells, while drug-delivering platforms can alter platelet and smooth muscle cell EV profiles through pharmacological effects [3,29]. We proposed that EVs can serve as biological templates for next-generation drug-eluting cardiovascular devices and offer a framework for understanding how biomaterial design can indirectly influence thrombo-inflammatory responses through vesicle-mediated communication networks [29].

Importantly, EVs and NET-related markers are also attractive candidates for monitoring and risk stratification. Both EV signatures and NET-related biomarkers (circulating dsDNA, CitH3, MPO-DNA complexes) can be sampled from blood, enabling non-invasive longitudinal monitoring of thrombo-inflammatory activity after device implantation [19,46]. Integration of such biomarkers into AI-enabled monitoring platforms (a concept discussed in more detail in Section 5) could enable the early detection of adverse biological trends before clinical deterioration occurs.

## 4. Taxonomy of Immune–Device Interaction Phenotypes

We then translated these mechanistic insights into a phenotype-based framework that links device classes to characteristic immune response patterns.

**Figure 2:**
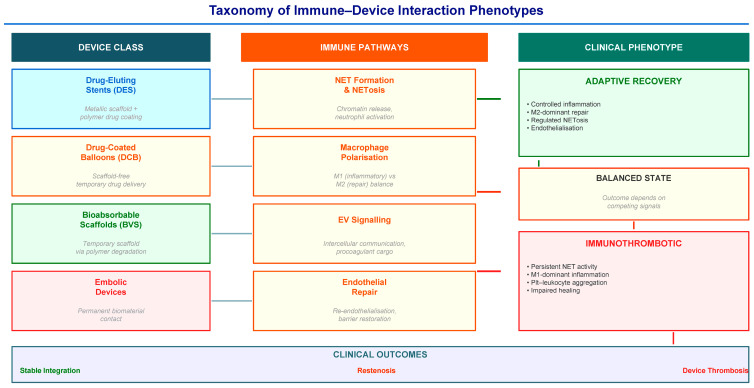
Conceptual taxonomy linking endovascular device classes to immune response phenotypes. Different classes of endovascular devices interact with host vascular tissues through different biomaterial and pharmacological properties. These interactions influence thrombo-inflammatory pathways, including neutrophil extracellular trap formation, macrophage polarization, extracellular vesicle signaling, and endothelial repair. The resulting biological trajectories range from adaptive recovery responses to persistent immunothrombotic states, ultimately shaping clinical outcomes such as stable integration, restenosis, or device-associated thrombosis. All arrows are solid lines indicating established or proposed directional relationships. Arrows from device classes to immune pathways indicate that each device type engages multiple immune mechanisms. Arrows from immune pathways to clinical phenotypes indicate the resulting biological trajectory. Arrows from phenotypes to clinical outcomes indicate the downstream clinical consequence.

Previous sections highlight that endovascular device outcomes arise from a complex interplay between biomaterial properties, vascular injury biology, and host immune responses. However, despite extensive evidence that immune mechanisms influence device performance, there is currently no systematic framework that maps immune response patterns to specific device classes. Here, we propose a taxonomy of immune–device interaction phenotypes that links device design variables to measurable thrombo-inflammatory trajectories (Figure 2).

### 4.1. Defining Immune–Device Interaction Phenotypes

An immune–device phenotype can be defined as a composite biological response arising from the interaction between implanted biomaterials and host immune systems, and measurable through cellular, molecular, and imaging biomarkers. Two broad trajectories can be distinguished: adaptive recovery phenotypes characterized by controlled inflammatory signaling, M2-dominant macrophage polarization, regulated NET formation, and progressive endothelialization [11,44]; and immunothrombotic phenotypes, characterized by persistent NET activity, M1-dominant inflammation, platelet-leukocyte aggregation, and impaired healing [10,12,15]. Between these endpoints lies a balanced state of inflammation where the outcome depends on the dynamic interaction of competing signals.

Clinical and pathological observations from coronary stent studies demonstrate the importance of such different healing responses. Histopathological analyses of drug-eluting stents have shown that delayed endothelialization, persistent fibrin deposition, and inflammatory cell infiltration are associated with late thrombotic events [1,3,43]. These findings suggest that the balance between adaptive and immunothrombotic phenotypes may determine whether a device will be successful.

### 4.2. Device Classes and Immune Response Signatures

Different classes of endovascular devices present different biomaterial and pharmacological variables that can influence immune–device phenotypes. Drug-eluting stents (DES) combine metallic scaffolds with the polymer-based drug delivery of antiproliferative agents. The permanent metallic platform provides continuous mechanical support but creates a persistent foreign body stimulus. For example, first-generation durable-polymer DES have been associated with delayed endothelialization and a delayed thrombotic risk; this demonstrates how sustained foreign body signaling can shift healing toward an immunothrombotic phenotype. The immune phenotype is characterized by an acute increase in NET triggered by bare metal contact and polymer inflammation, followed by suppression of drug-mediated smooth muscle proliferation as well as delayed endothelialization [1,3,43]. Dominant failure modes include late and very late stent thrombosis and neoatherosclerosis [43]. The main device categories and typical immune response signatures are summarized in Table 1.

Drug-coated balloons (DCBs) represent a different paradigm where temporary drug delivery occurs without a permanent scaffold. In contrast, DBCs avoid chronic scaffold-related inflammation, but endothelial toxicity and variability in drug transfer efficiency can still contribute to restenosis or maladaptive remodeling. While DCBs avoid prolonged foreign body exposure, drug transfer efficiency and adjuvant effects reveal their own immunological variables [5,6,7]. The duration of the NET response is expected to be shorter because there is no continuous biomaterial contact, but the cytotoxic effects of paclitaxel on endothelial cells can disrupt the anti-inflammatory EV secretome [29]. The dominant failure modes involve elastic rebound and negative remodeling rather than neointimal hyperplasia.

Bioabsorbable vascular scaffolds (BVSs) introduce temporal complexity. Clinical experience with BVSs provides a concrete example of temporally evolving immune responses, with early implantation injury followed by an inflammatory phase associated with subsequent degradation. Initial implantation triggers an acute inflammatory response similar to metallic stents, but the degradation phase (12–36 months) introduces a second inflammatory wave as the polymer fragments activate the inflammation signal and alter the local pH [4,21]. This two-phase immune trajectory distinguishes BVSs from other device classes and creates a long-term fragility window. Clinical experience has confirmed that scaffold thrombosis occurs at higher than expected rates during the degradation phase [4]. Embolization systems and other endovascular technologies, including liquid embolic agents, trigger immune responses through biomaterial contact and local inflammatory signaling. While these devices differ significantly in their design purposes, they share a fundamental characteristic: triggering thrombo-inflammatory cascades that influence clinical outcomes.

### 4.3. Biomarkers of Immune–Device Phenotypes

Operationalizing the immune–device phenotype concept requires measurable biomarkers that can capture thrombo-inflammatory activity after device implantation. Candidate biomarker classes include: NET-associated markers reflecting neutrophil activation (circulating dsDNA, CitH3, MPO-DNA complexes) [19,20,49]; EV signatures reflecting intercellular signaling at the device interface (exomal miRNA profiles, EV-dependent tissue factor) [22,46]; macrophage polarization markers reflecting M1/M2 balance (soluble CD163, CCL18) [32]; and standard inflammatory indices providing general inflammatory context (CRP, IL-6, IL-1β) [11,40].

Importantly, these biomarkers should be interpreted not in isolation, but as components of integrated biological signatures that combine immune markers with imaging findings and clinical parameters. As the taxonomy presented here suggests, the optimal panel composition and measurement timing will likely be device-class specific. A range of circulating and cellular biomarkers can capture thrombo-inflammatory activity associated with endovascular device implantation. Representative biomarker classes and their potential clinical implications are summarized in Table 2.

### 4.4. Implications for Immunologically Adaptive Device Strategies

The proposed taxonomy has several implications for endovascular device development and clinical management. First, it emphasizes that device performance may depend on patient-specific immune profiles as well as device design. Second, it suggests that monitoring strategies should be tailored to the expected immune trajectory of each device class. Third, it identifies potential therapeutic targets (NET formation, macrophage polarization, EV-mediated signaling) that can be modulated through next-generation device coatings or adjuvant therapies [21,29,30]. By framing endovascular interventions through the lens of immune–device phenotypes, this taxonomy provides a foundation for an immunologically adaptable device paradigm.

## 5. AI-Enabled Life Cycle Monitoring and Closed-Loop Intervention

Finally, we consider how these measurable immune phenotypes can be operationalized within AI-enabled monitoring and adaptive clinical decision-making processes.

Advances in vascular immunology and biomaterials research increasingly demonstrate that endovascular device performance stems from a dynamic interplay between device characteristics and host biological responses. Capturing this dynamic requires monitoring strategies that extend beyond the current paradigm of periodic clinical assessment. AI offers a computational framework that can integrate multimodal data streams, detect patterns unseen in traditional analysis, and support adaptive clinical decision-making [31,32,33].

Traditional post-implantation surveillance relies on periodic imaging and clinical assessment at predefined intervals. While basic, these approaches only capture snapshots of a constantly evolving biological process and may miss early adverse trends. Real-world evidence generated through clinical records and electronic health records provides richer longitudinal data, but has traditionally been analyzed retrospectively rather than used for real-time prediction [34,50].

AI methods are becoming increasingly capable of integrating heterogeneous clinical data streams to detect subtle patterns preceding overt clinical events. Machine learning models can combine structured variables from electronic health records, unstructured imaging data, laboratory results, and where available, wearable sensor signals [31,35].

Within the context of immunologically adaptive devices, AI-monitoring systems may incorporate multimodal biological signals reflecting thrombo-inflammatory activity and vascular healing. Candidate inputs include NET-associated biomarkers (dsDNA, CitH3, MPO-DNA), EV signatures, inflammatory cytokines, intravascular imaging features (IVUS, OCT), and standard clinical parameters. Device-class-specific models based on the immunity–device phenotype taxonomy (Section 4) can weight these inputs according to the expected immune trajectory for each device type. AI-driven decision-making can guide intravascular device selection in aortic disease [21], which provides a basis for the monitoring architecture described here.

**Figure 3:**
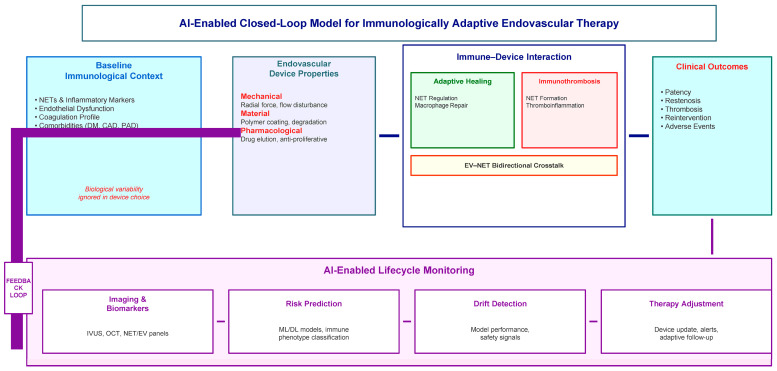
Closed-loop monitoring framework for immunologically adaptive endovascular devices. This schematic illustrates a conceptual closed-loop monitoring architecture integrating device characteristics, immune biomarkers, and clinical data streams. The patient’s immune status, thrombo-inflammatory biomarkers (including NET-related markers and extracellular vesicle signatures), imaging findings, and clinical variables are continuously assessed using AI-based predictive models. Model outputs are compared to expected recovery trajectories for a given device class and patient profile, providing adaptive recommendations for follow-up, risk management, and potential intervention.

A key feature of such monitoring systems is their ability to operate within a closed-loop clinical framework (Figure 3). In engineering terms, a closed-loop system continuously measures outputs, compares them to predicted trajectories, and adjusts inputs accordingly. When applied to endovascular therapy, this means that AI models continuously evaluate thrombo-inflammatory biomarkers and clinical signals, compare them to expected recovery trajectories for a specific device class and patient immune profile, and generate adaptive recommendations, including updated device selection guidance for treatment adjustment, follow-up planning, safety alerts, and subsequent interventions.

Recent studies on medical AI applications highlight that successful clinical implementation requires life cycle management, including monitoring model performance after implementation. Machine learning models can degrade over time through distribution shifts, population changes, or evolving clinical practices [32,33,37]. Therefore, shift detection algorithms that identify when model predictions deviate from observed outcomes are essential components of any AI-enabled monitoring system. This oversight layer addresses a critical gap in current device monitoring frameworks that rely on static analyses rather than adaptive, real-time monitoring.

AI frameworks can also enable the integration of population-level evidence with individual patient monitoring. Large clinical registries and real-world datasets can create a learning system that evolves with increasing data, improving predictive models over time [34,51]. Post-market monitoring can shift from retrospective safety reviews to proactive, AI-enabled signal detection and support evolving regulatory expectations for medical device life cycle monitoring [36,37]. At a practical level, such an architecture would include four functional modules: data collection (biomarkers, imaging, electronic health records), feature integration, risk estimation and deviation detection, and clinical output generation for follow-up planning, warning, and treatment adjustment. The increasing number of FDA-approved AI-powered medical devices and emerging regulatory guidelines for adaptive algorithms support the feasibility of this architecture.

Importantly, AI-enabled monitoring should complement, not replace, the mechanistic understanding of vascular biology. Prediction algorithms are most effective when based on biologically significant variables identified through the frameworks described in Section 2, Section 3 and Section 4. The integration of immune biomarkers into AI-risk models represents a critical link between biological understanding and computational architecture, capturing the immunological dimension that explains inter-patient variability in device outcomes.

## 6. Life Cycle Evidence Framework for Immunologically Adaptive Devices

**Figure 4:**
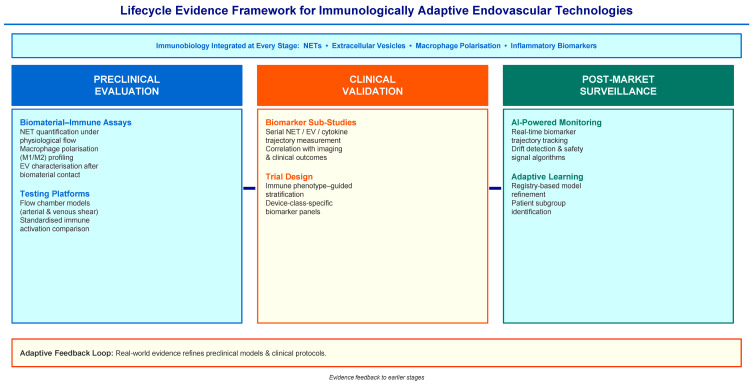
Life cycle evidence framework for immunologically adaptive endovascular technologies. This framework expands traditional medical device evaluation by integrating immunobiology into the entire device life cycle. Preclinical studies include biomaterial–immune interaction assays such as NET formation, macrophage polarization, and extracellular vesicle profiling under flow conditions. Clinical trials include biomarker-focused sub-studies that visualize immune responses and correlate them with clinical outcomes. Post-marketing surveillance integrates registries and AI-enabled monitoring systems to detect safety signals, improve predictive models, and support adaptive life cycle management. All arrows are solid lines. Forward arrows indicate the progression from preclinical evaluation through clinical validation to post-market Surveillance. The feedback arrow at the bottom indicates the adaptive evidence loop in which real-world data refines preclinical models and clinical protocols.

The concept of immunologically adaptive endovascular devices raises new requirements for how device performance should be assessed throughout development and clinical application (Figure 4). The minimum evidence for an immunologically adaptable device platform should include: (i) reproducible preclinical immunoprofiling assays, (ii) biomarker-linked clinical outcome relationships, (iii) external validation of monitoring algorithms, and (iv) post-market surveillance processes capable of detecting safety signals and model deviation. Traditional medical device evaluation follows a linear path from bench testing to clinical trials and post-market surveillance, with mechanical performance and clinical efficacy endpoints being primarily assessed at each stage [34,51]. The framework proposed here extends this model to include thrombo-inflammatory biology, immune biomarkers, and AI-assisted monitoring at each stage.

In the preclinical phase, biomaterial evaluation has historically focused on mechanical stability, degradation kinetics, and cytocompatibility. The increasing recognition of the immune–device interface suggests that preclinical testing should include systematic immune profiling: NET quantification under physiological flow conditions, macrophage polarization assays in response to device materials and degradation products, and EV characterization after biomaterial contact [39,48]. Flow chamber models exposing human neutrophils to device surfaces under arterial and venous shear conditions will provide standardized platforms for comparing immune activation across device classes. Clinical validation represents the next stage in the evidence pathway. Randomized controlled trials are still important for assessing device safety and efficacy, but many thrombo-inflammatory processes affecting long-term outcomes are not captured by conventional study endpoints. Biomarker sub-studies integrated into device studies can measure serial NET, EV, and inflammatory marker trajectories and correlate immune profiles with imaging and clinical outcomes [19,46,49]. Such studies will test whether immune–device phenotypes predict clinical trajectories beyond what standard risk factors capture. The third component involves the generation of real-world evidence and post-market monitoring. Medical devices often continue to evolve after regulatory approval through design iterations, new indications, and expanded patient populations [34,51]. In the immunologically adaptive paradigm, real-world monitoring gains add importance because biological responses can vary across patients and clinical contexts. AI-enabled surveillance systems that continuously track biomarker trajectories and clinical outcomes in device registries can detect emerging safety signals, identify patient subgroups with different recovery responses, and refine predictive models over time [33,36,37].

Together, these stages define a closed-loop life cycle evidence model for immunologically adaptive endovascular technologies. Preclinical testing provides a mechanistic perspective on immune–device interactions; clinical trials validate biomarker-guided predictions in controlled settings; and real-world monitoring sustains adaptive learning after deployment. This framework ensures that immune biology is integrated into the evidence base at every stage of device evaluation, rather than as an afterthought.

## 7. Testable Predictions and Research Roadmap

The framework proposed in this study suggests that endovascular device performance arises from a dynamic interplay between biomaterial properties, thrombo-inflammatory biology, and patient-specific immune responses. For this conceptual model to go beyond descriptive appeal, it needs to generate specific, falsifiable predictions that can be experimentally and clinically tested.

**Prediction 1**: Key thrombo-inflammatory biomarkers will predict post-intervention device outcomes. Circulating markers associated with NET formation (dsDNA, CitH3, MPO-DNA), endothelial activation, or extracellular vesicle profiles measured before endovascular intervention should provide independent predictive information beyond established clinical risk factors [15,19,49]. *Test*: A prospective cohort study measuring pre-procedure NET and EV panels in patients undergoing coronary or peripheral intervention, with primary endpoints being 12-month binary restenosis and major adverse cardiovascular events.

**Prediction 2**: Device design parameters influence thrombo-inflammatory signaling profiles. Variations in surface chemistry, polymer coatings, or drug delivery kinetics should modulate immune activation in device-specific patterns consistent with phenotype taxonomy [1,3,38]. *Test*: Direct comparison of serial NET and inflammatory biomarker profiles in patients randomized to DES, DCB, and BVS in paired lesion settings.

**Prediction 3**: Device surface properties modulate NET formation under physiological shear conditions [8,10,48]. *Test*: Quantification of NET formation by in vitro parallel plate flow chamber experiments exposing human neutrophils to device material samples under arterial and venous shear, fluorescence microscopy, and ELISA for NET-associated markers.

**Prediction 4**: AI models integrating immune biomarkers will outperform models based solely on clinical variables in predicting adverse events after device implantation [31,35,38]. Primary model comparison criteria should include discrimination in decision curve analysis, calibration, and clinically significant net utility. *Test*: Retrospective model development using registry data, comparison of model AUC, calibration, and net reclassification improvements with stored biological samples with and without NET/EV biomarker inputs, followed by prospective external validation.

**Prediction 5**: Closed-loop AI-driven monitoring will enable earlier detection of device failure signals compared to standard fixed-program monitoring [36,37]. Secondary assessment points may include re-intervention time, number of unscheduled imaging studies, and false alarm rate. *Test*: Pragmatic trial comparing the time to detection of clinically significant restenosis or thrombosis between adaptive AI-driven monitoring and traditional protocol-driven monitoring programs.

Translation roadmap

**Short-term (1–2 years)**: Develop in vitro testing platforms for device-specific NET induction under flow conditions; validate candidate biomarker panels in existing cohorts with stored biological samples; develop and internally validate AI-risk models incorporating immune biomarkers.

**Medium-term (3–5 years)**: Conduct prospective biomarker-driven clinical pilot studies comparing immunologically classified management with traditional management; externally validate AI models across multiple device registries; collaborate with regulatory bodies on adaptive monitoring frameworks for AI-enabled devices.

**Figure 5:**
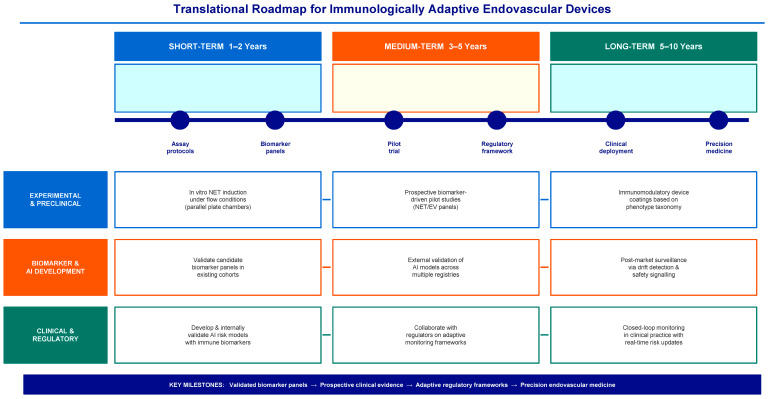
Translational roadmap for immunologically adaptive endovascular devices. The roadmap outlines a phased strategy for translating immunologically sensitive device concepts into clinical practice. Short-term research focuses on experimental models of NET induction under physiological flow conditions, validation of immune biomarker panels, and development of preliminary AI predictive models. Medium-term studies include prospective biomarker-guided clinical trials and registry-based validation of AI-monitoring systems. Long-term goals include the implementation of closed-loop monitoring systems, immunomodulatory device designs, and adaptive regulatory surveillance frameworks. All arrows are solid lines indicating the forward progression of research activities from short-term to long-term goals. Horizontal arrows between columns represent temporal progression within each research track.

**Long-term (5–10 years)**: Establish closed-loop monitoring systems in clinical practice with real-time risk updates; develop immunomodulatory device coatings based on phenotype taxonomy; establish post-market surveillance platforms using drift detection and safety signaling algorithms as standard practice. The proposed translational pathway for the development of immunologically adaptive endovascular technologies is summarized in Figure 5.

Several key barriers that may limit the clinical translational process include the lack of standardized NET tests, variability in EV measurement methods, limited multimodal datasets, and regulatory uncertainty surrounding adaptive AI systems. Potential solutions include multi-center biomarker harmonization efforts, consensus testing protocols, prospective registry-linked biobanks, and early collaboration with regulatory bodies during algorithm development.

Despite the conceptual promise of immunologically adaptable endovascular devices, several translation challenges must be addressed before they can be implemented in clinical practice. The main challenges and potential solutions are summarized in Table 3.

## 8. Outlook: Toward Precision Endovascular Bioengineering

Advances in vascular immunology, biomaterial science, and digital health technologies are converging to reshape how cardiovascular devices are conceptualized, designed, and monitored. The framework outlined in this perspective integrates thrombo-inflammatory biology, immune–device phenotype taxonomy, AI-powered life cycle monitoring, and a structured evidence pathway into a coherent paradigm for next-generation endovascular intervention. In this emerging model, precision endovascular treatment will likely depend on the incorporation of patient-specific biological information into device design and selection. Immune biomarkers reflecting thrombo-inflammatory status, including NET burden, EV signatures, and macrophage polarization profiles, can complement traditional risk factors in guiding device selection, adjuvant therapy, and monitoring intensity [15,20,29,38]. Over time, the accumulation of real-world evidence from AI-powered monitoring can support personalized treatment algorithms that match patients with device classes most likely to produce favorable immune–device interactions.

Realizing this vision will require closer integration of biomaterial engineering, vascular biology, and data science. Device development processes may increasingly include immunological screening alongside traditional mechanistic and pharmacological testing [39,48]. Clinical trials should also include biomarker-focused sub-studies that capture thrombo-inflammatory dynamics, rather than relying solely on binary endpoints [19,46]. In parallel, AI model development should rely on mechanistic understanding and ensure that prediction systems reflect the biological reality of immune–device interactions, rather than relying solely on pattern recognition [31,35].

As these technologies mature, regulatory frameworks for cardiovascular devices may also evolve. Traditional approval pathways emphasize safety and efficacy endpoints measured during pre-market evaluation. In contrast, the life cycle monitoring paradigm proposed here incorporates AI systems that continuously evaluate device performance, detect safety signals, and adapt to changing patient populations. This approach is consistent with emerging regulatory models for adaptive algorithms, including life cycle-based approaches to predefined change control schemes and real-world evidence [34,36,37]. Transparency reporting and standardization of NET-related measures will be practical priorities to accelerate regulatory adoption of AI-powered monitoring systems [33,49].

In conclusion, the concept of immunologically adaptive endovascular devices highlights the opportunity to move beyond static device paradigms and advance toward therapeutic systems that continuously respond to the patient biology. By proposing a unified framework that includes testable predictions, a practical taxonomy, and a translation roadmap, we aim to foster interdisciplinary collaboration and steer the next generation of cardiovascular devices toward truly personalized, adaptive, and immunologically informed intervention. Future studies integrating vascular immunology, biomaterial science, and adaptive computational monitoring will determine whether this paradigm can transform endovascular therapy from a device-centric intervention to a biologically responsive treatment system.

## Figures and Tables

**Table 1:** Immune response profiles associated with major classes of endovascular devices.

Device Class	Key Design Features	Dominant Immune Response	Typical Failure Modes
Drug-eluting stents	Metallic scaffold + polymer drug coating	Early NET activation, delayed endothelialization	Late-stage stent thrombosis, neoatherosclerosis
Drug-coated balloons	Transient drug delivery without permanent scaffold	Shorter inflammatory response but endothelial toxicity	Elastic retraction, restenosis
Bioabsorbable vascular scaffolds	Temporary scaffold via polymer degradation	Biphasic inflammation during degradation	Scaffold thrombosis
Embolic devices	Permanent biomaterial contact	Local inflammatory response and thrombosis	Device-related thrombosis

**Table 2:** Major immuno-biomarkers relevant to endovascular device responses.

Biomarker Category	Examples of Biomarkers	Biological Meaning	Potential Clinical Application
NET-related markers	dsDNA, CitH3, MPO–DNA complexes	Neutrophil activation and NET formation	Prediction of thrombotic risk and restenosis
Extracellular vesicle signatures	EV-associated miRNAs, EV tissue factor	Intercellular signaling and coagulation activity	Monitoring of thrombo-inflammatory signaling
Macrophage polarization markers	CD163, CCL18	M1/M2 immune balance during vascular healing	Identification of adaptive and inflammatory repair
Inflammatory cytokines	CRP, IL-6, IL-1β	Systemic inflammatory activity	Contextualization of immune system responses

Abbreviations: dsDNA, double-stranded DNA; CitH3, citrullinated histone H3; MPO–DNA, myeloperoxidase–DNA complexes; NET, neutrophil extracellular trap; EV, extracellular vesicle; miRNA, microRNA; CD163, cluster of differentiation 163; CCL18, C–C motif chemokine ligand 18; CRP, C-reactive protein; IL-6, interleukin 6; IL-1β, interleukin 1 beta.

**Table 3:** Key translation challenges and proposed solutions for immunologically adaptive endovascular devices.

**Challenge**	**Why It Matters**	**Proposed Solutions**
Lack of standardized NET assays	Variability in NET measurements (dsDNA, CitH3, MPO-DNA) limits reproducibility across studies and clinical settings	Develop consensus protocols for NET quantification under standardized flow conditions and sample processing protocols; establish reference ranges in multicenter studies
Heterogeneity in extracellular vesicle (EV) characterization	Differences in EV isolation and analysis methods reduce comparability and clinical applicability	Adopt standardized EV isolation and reporting guidelines; integrate multi-omics profiling to identify reproducible EV signatures
Limited linkage between biomarkers and clinical outcomes	Immune biomarkers are often studied in isolation without robust association to device-specific outcomes	Conduct prospective biomarker-focused studies correlating NET/EV profiles with restenosis, thrombosis, and imaging outcomes
Fragmented multimodal datasets	Clinical, imaging, and biomarker data are rarely integrated, limiting development of predictive models	Establish integrated registries combining EHR, imaging, and biomarker data; promote interoperable data standards
AI model drift and generalizability issues	Predictive models may lose accuracy over time due to changes in populations or clinical practice	Implement continuous model monitoring, deviation detection algorithms, and periodic recalibration using real-world data
Regulatory uncertainty for adaptive AI systems	Traditional regulatory pathways are not fully compatible with continuously learning algorithms	Develop regulatory frameworks that include predefined change control plans and life cycle monitoring strategies
Integration into clinical workflows	Complex monitoring systems may not be easily adopted in routine practice	Design user-centered clinical interfaces and decision support tools that integrate seamlessly with existing workflows

## Data Availability

No new data were created or analyzed in this study. Data sharing is not applicable to this review article.
